# Supplementary material for: PHLOWER leverages single-cell multimodal data to infer complex, multi-branching cell differentiation trajectories
Source: Nat Methods. 2025 Oct 23;22(11):2328–36. doi: 10.1038/s41592-025-02870-5 (PMC12615267; doi:10.1038/s41592-025-02870-5)
Supplement: Supplementary file 1 — Supplementary Figs. 1–10 and Supplementary Tables 1 and 2. [file 41592_2025_2870_MOESM1_ESM.pdf]

nature methods

Supplementary information https://doi.org/10.1038/s41592-025-02870-5

PHLOWER leverages single-cell multimodal data to infer complex, multi-branching cell differentiation trajectories

In the format provided by the authors and unedited

List of Figures

S1 PHLOWER workflow for the simulated tree dataset with 10 total branches 2
S2 Pancreas trajectory for all evaluated methods 3
S3 Neurogenesis trajectory for all evaluated methods 4
S4 Spatial expression of cell type specific transcription factors for day 19 and day 25 kidney organoids . 5
S5 Spatial gene expression for cell type specific markers for day 19 and day 25 kidney organoids 6
S6 Spatial gene expression for cell type specific transcription factors for scrambled siRNA and siRNA treated 7
S7 Spatial gene expression for cell type specific markers for scrambled siRNA and siRNA treated 8
S8 0-dimensional and 1-dimensional cycles 9
S9 Example of an ordered simplicial complex and boundary maps B_1 and B_2 representing this complex . 9
S10 Schematic showing how to create a tree using edges lay in cumulative trajectory space 10

List of Tables

S1 List of all Dynverse data sets with a tree structure sorted by the number of branches 11
S2 List of genes used in the Xenium spatial profiling panel 12

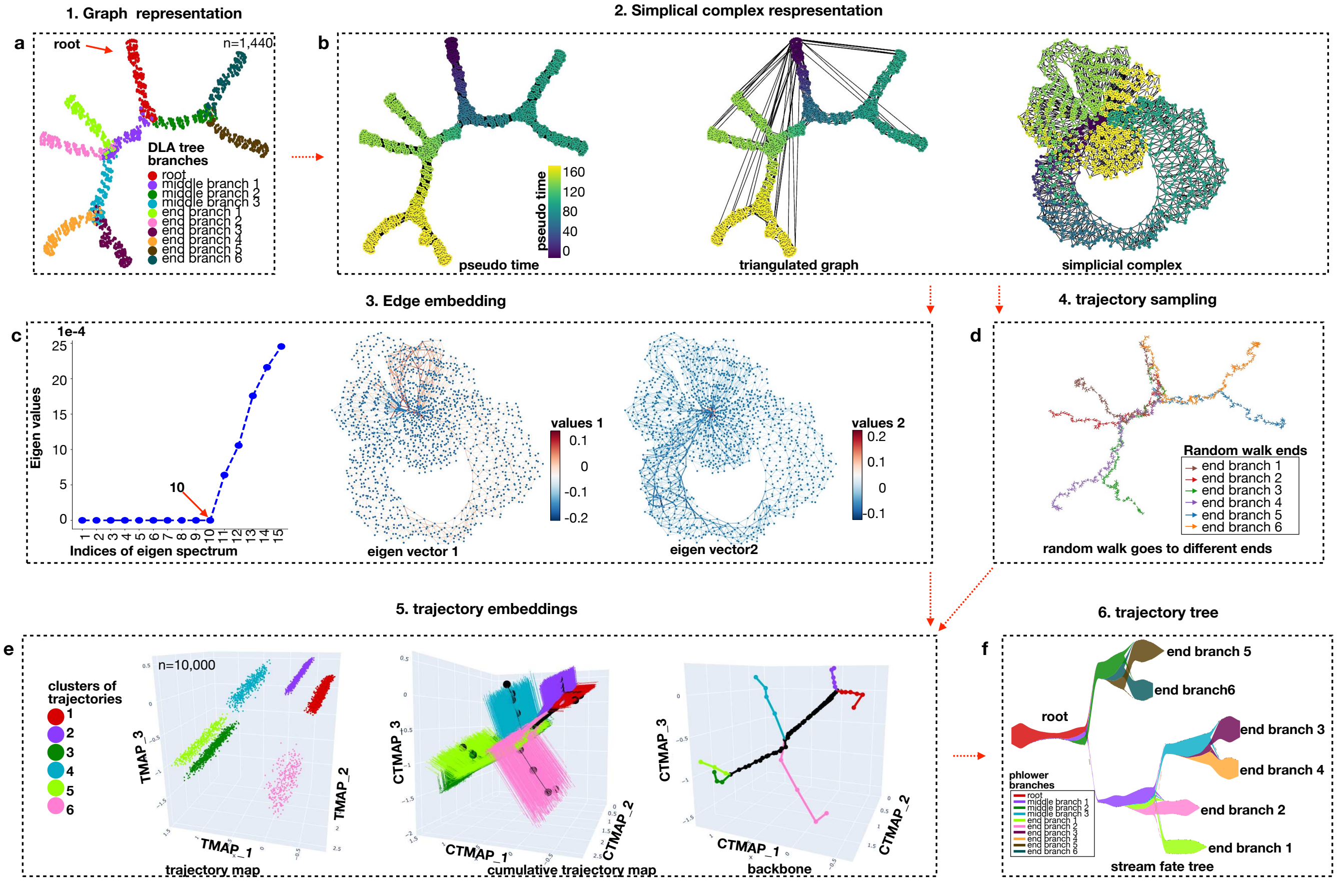

Figure S1. PHLOWER workflow for the simulated tree dataset with 10 total branches Panels **a-f,** show distinct PHLOWER estimates for the simulated data with 10 branches exactly as described in Extended Data Fig.1. Note that in this complex multi-branched data, the 2 dimensional representation of the graph layout of the simplicial complex (**b**) can not capture the data complexity and does not properly display all the holes. Clustering analysis in the trajectory embedding detects the six main trajectories present in the data (**e**) and recovers an almost perfect differentiation tree (**f**). Of note, the number of zero-eigenvalues (10) is higher than the number of main trajectories (6). This is potentially due to small (and noise associated) holes in the graph. This do not impact the precision of the clustering analysis to find the correct branches.

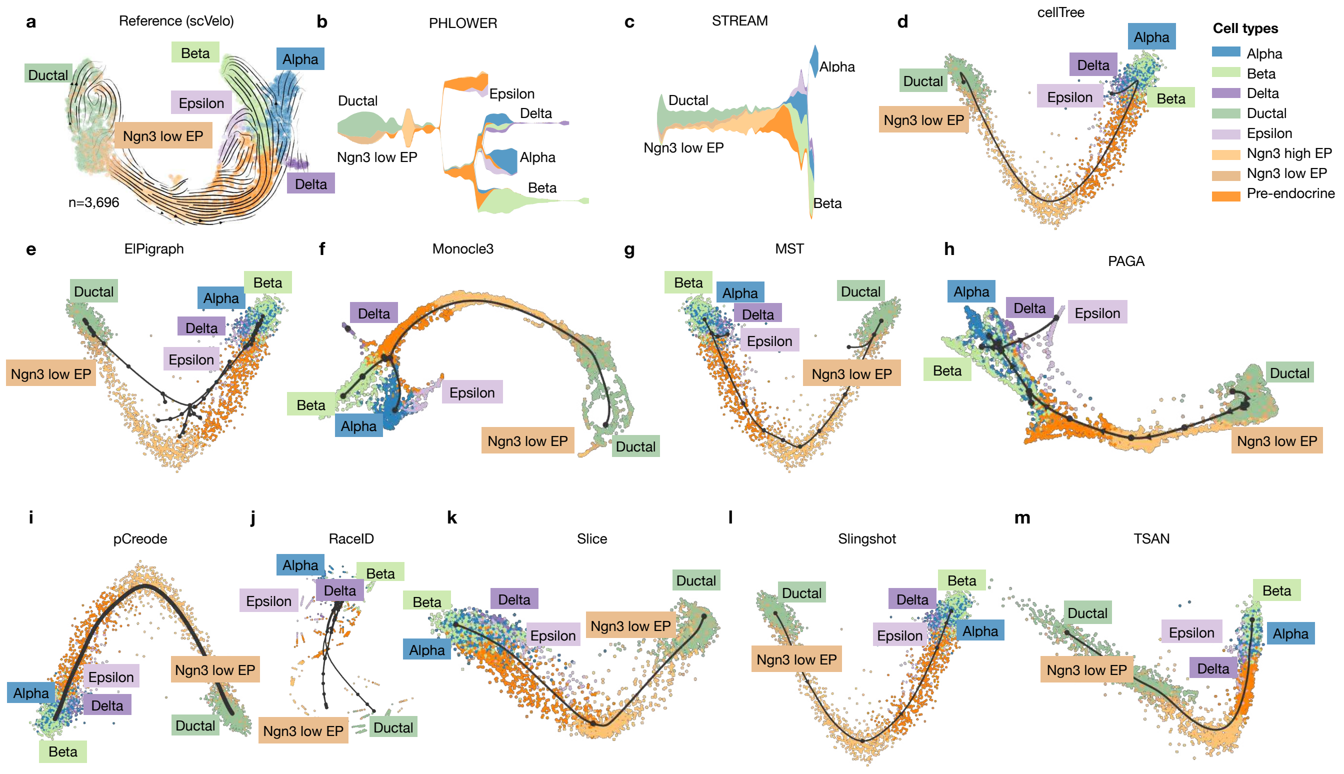

Figure S2. Pancreas trajectory for all evaluated methods. a, Displays the original embedding of the pancreas progenitor data (n=3,396) as reported in scVelo[1]. Colors corresponds to the cell annotation provided in[1]. This information is not provided to algorithms. **b-m,** shows the results for all evaluated methods. We use the default embedding of the tool for visualization. For several approaches (Slingshot, MST, pCreode,CellTree and ElpiGraph), this is based on PCA as implemented in Dynverse.

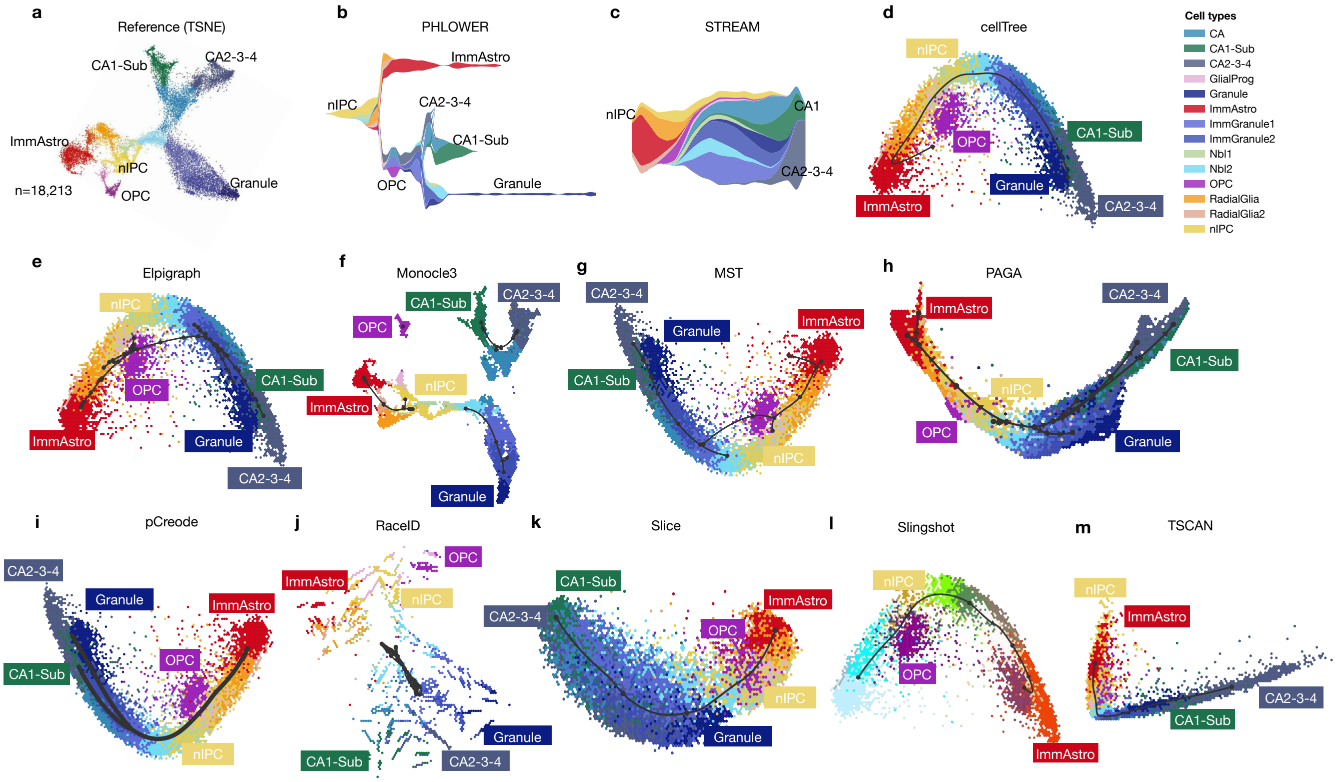

Figure S3. Neurogenesis trajectory for all evaluated methods. a, Displays the original embedding of the neurogenesis data (n=18,213) as reported in [2]. Colors corresponds to the cell annotation provided in[2]. This information is not provided to algorithms. **b-m,** shows the results for all evaluated methods.

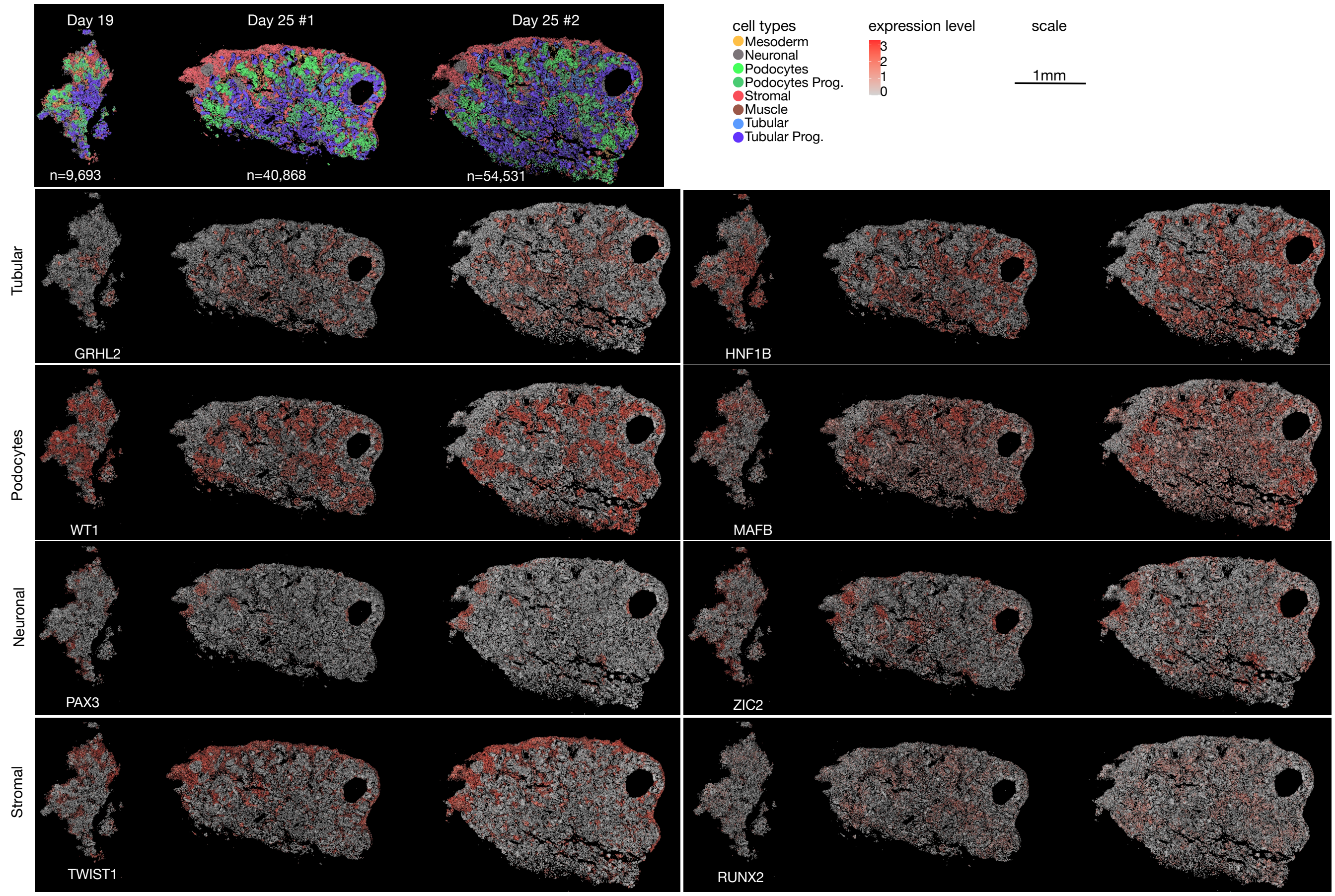

Figure S4. Spatial expression of cell type specific transcription factors for day 19 and day 25 kidney organoids.

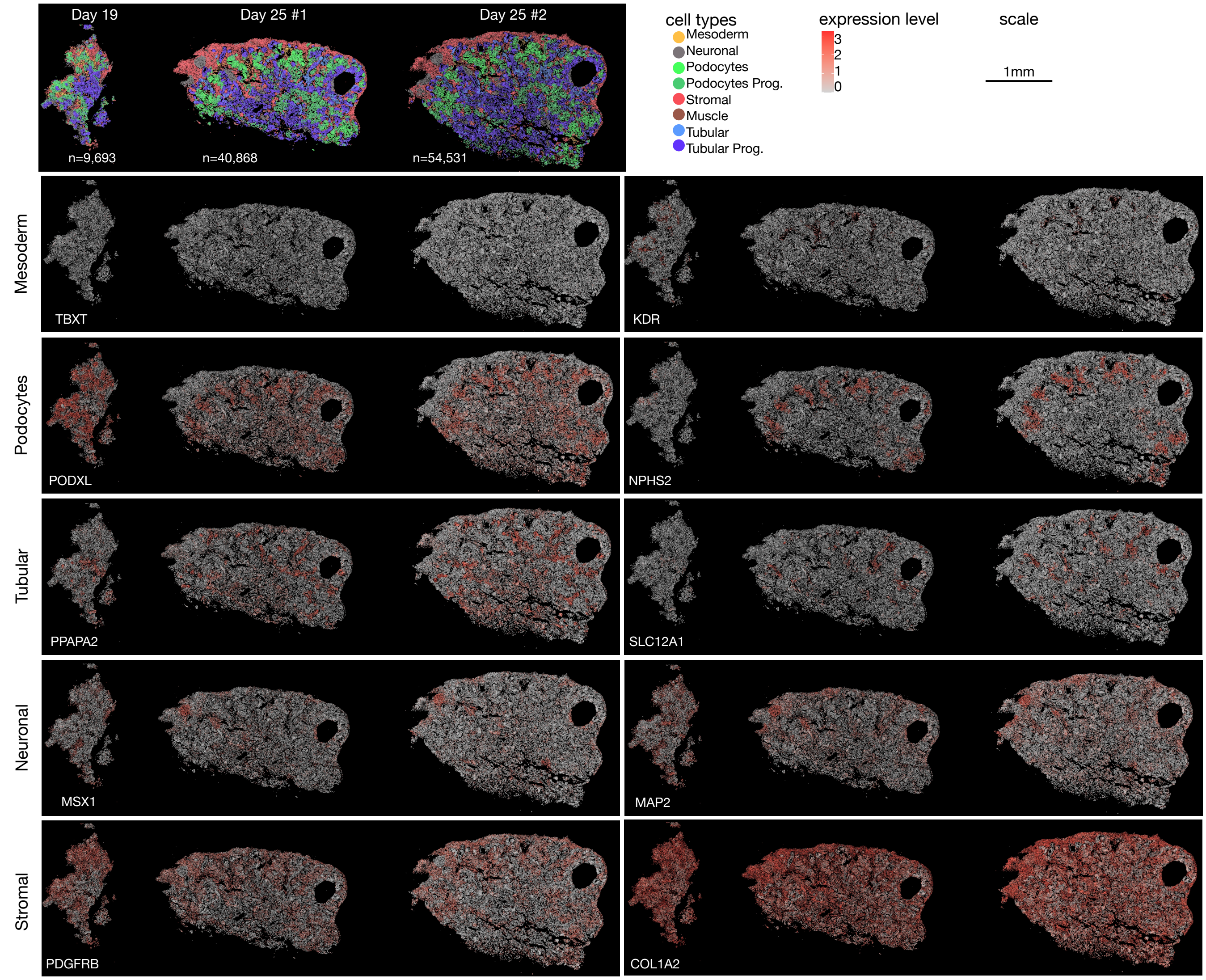

Figure S5. Spatial gene expression for cell type specific markers for day 19 and day 25 kidney organoids.

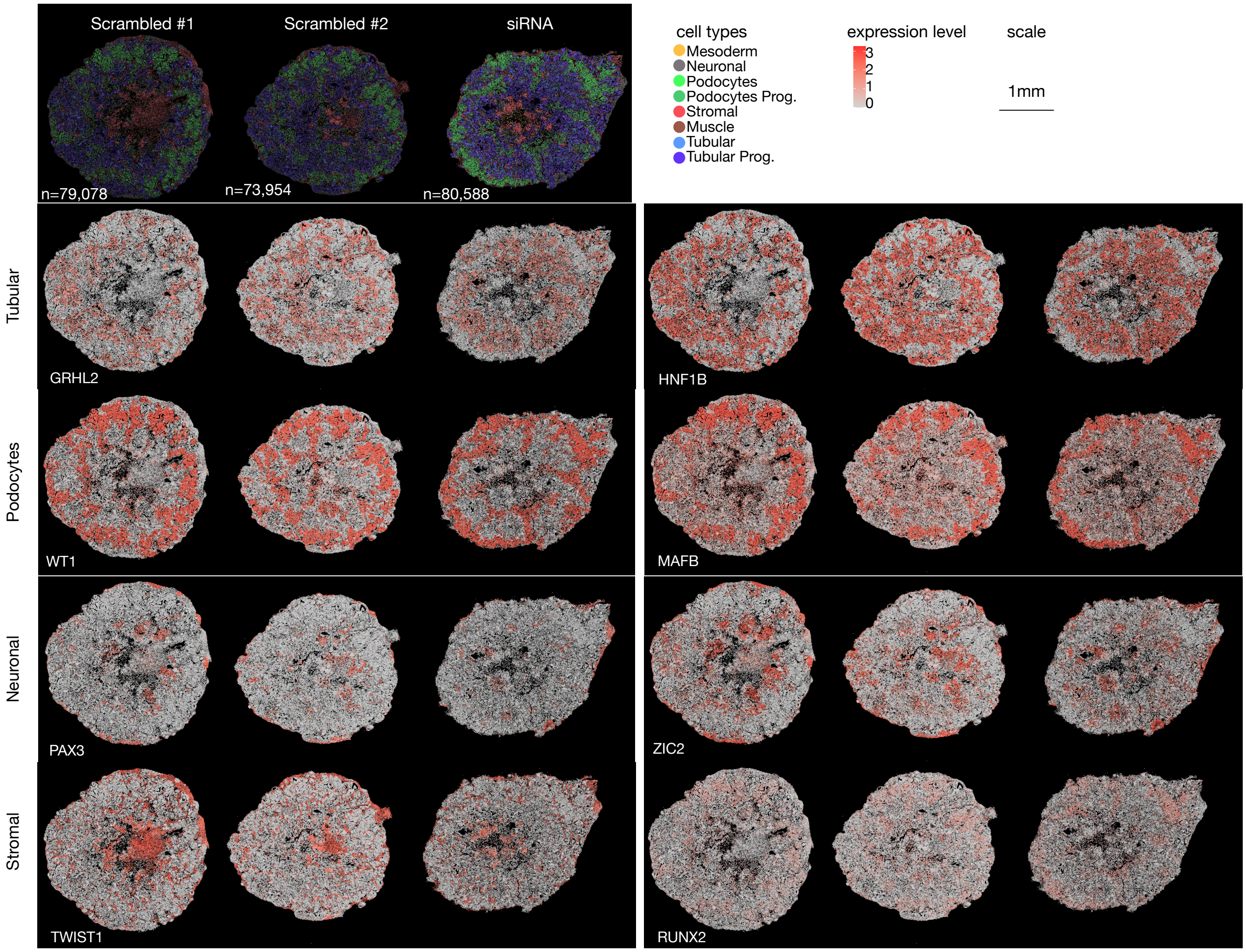

Figure S6. Spatial gene expression for cell type specific transcription factors for scrambled siRNA and siRNA treated.

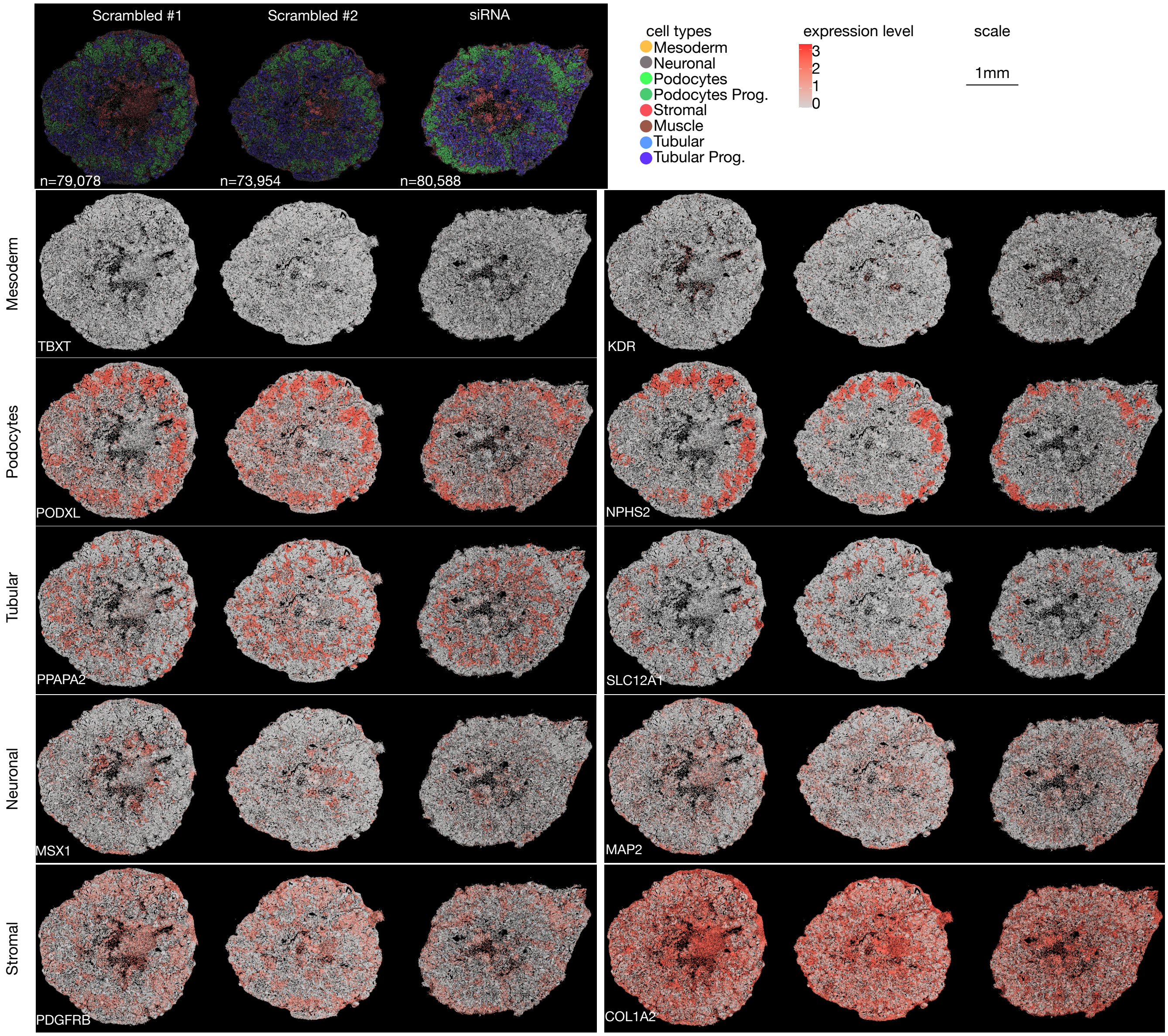

Figure S7. Spatial gene expression for cell type specific markers for scrambled siRNA and siRNA treated.

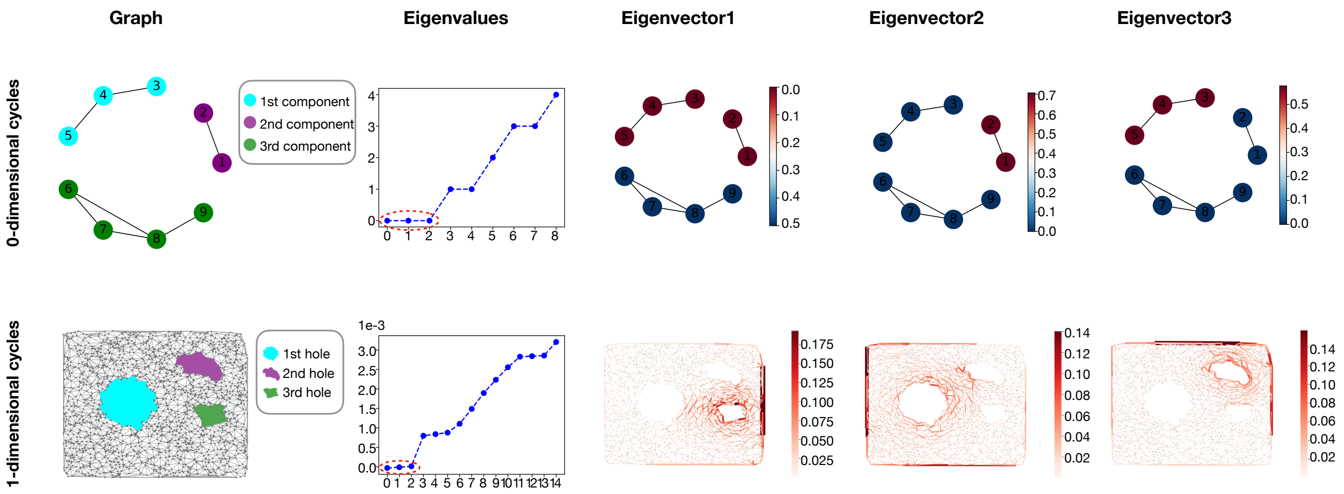

Figure S8. 0-dimensional and 1-dimensional cycles. We demonstrate the detection of 0-dimensional cycles (connected components) and 1-dimensional cycles (holes)[3] using the Graph Laplacian and the first-order Hodge Laplacian. In the first column, the upper side displays a graph with three 0-dimensional cycles (connected components), while the bottom side shows a simplicial complex with three 1-dimensional cycles (holes). In the second column, we observe the harmonic eigenvectors of the Hodge Laplacian on both the 1-simplicial complex (graph) and the 2-simplicial complex, each with three zero eigenvalues (up to numerical artifacts). The third, fourth and fifth columns depict these *harmonic* eigenvectors with zero-eigenvalues. We note that the eigenvectors of the graph Laplacian will be indexed over the nodes, whereas eigenvectors of the first Hodge Laplacian will be indexed over oriented edges. Signals of each eigenvector delineates nodes of distinct connected components (top) or contribution of edges to harmonic flows around holes (bottom) in the graph or simplicial complex.

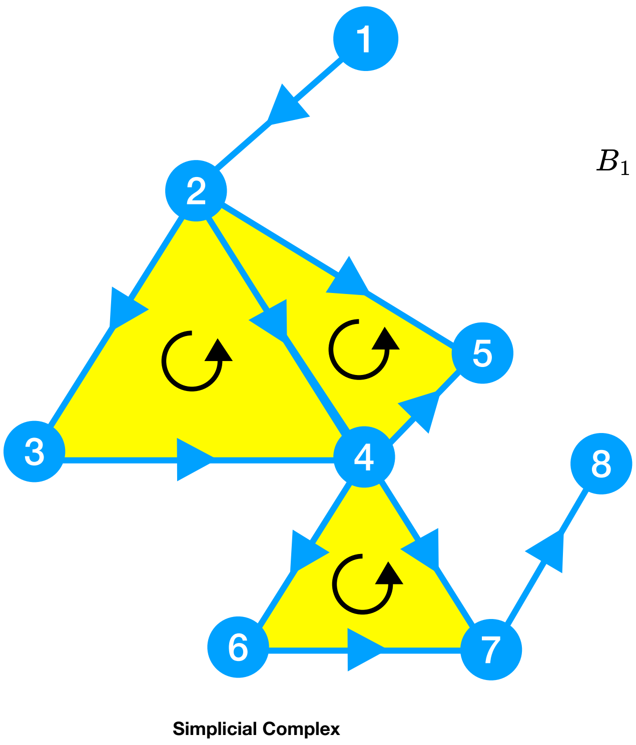

$B_1 =$

	[1, 2]	[2, 3]	[2, 4]	[2, 5]	[3, 4]	[4, 5]	[4, 6]	[4, 7]	[6, 7]	[7, 8]
1	−1	0	0	0	0	0	0	0	0	0
2	1	−1	−1	−1	0	0	0	0	0	0
3	0	1	0	0	−1	0	0	0	0	0
4	0	0	1	0	1	−1	−1	−1	0	0
5	0	0	0	1	0	1	0	0	0	0
6	0	0	0	0	0	0	1	0	−1	0
7	0	0	0	0	0	0	0	1	1	−1
8	0	0	0	0	0	0	0	0	0	1

$B_2 =$

	[2, 3, 4]	[2, 4, 5]	[4, 6, 7]
[1, 2]	0	0	0
[2, 3]	1	0	0
[2, 4]	−1	1	0
[2, 5]	0	−1	0
[3, 4]	1	0	0
[4, 5]	0	1	0
[4, 6]	0	0	1
[4, 7]	0	0	−1
[6, 7]	0	0	1
[7, 8]	0	0	0

Figure S9. Example of an ordered simplicial complex and boundary maps B_1 and B_2 representing this complex. Note that the order of edges/triangles correspond to a simple bookkeeping procedure, i.e. edge orientations are in the direction of increasing vertex index. The same applies for triangles.

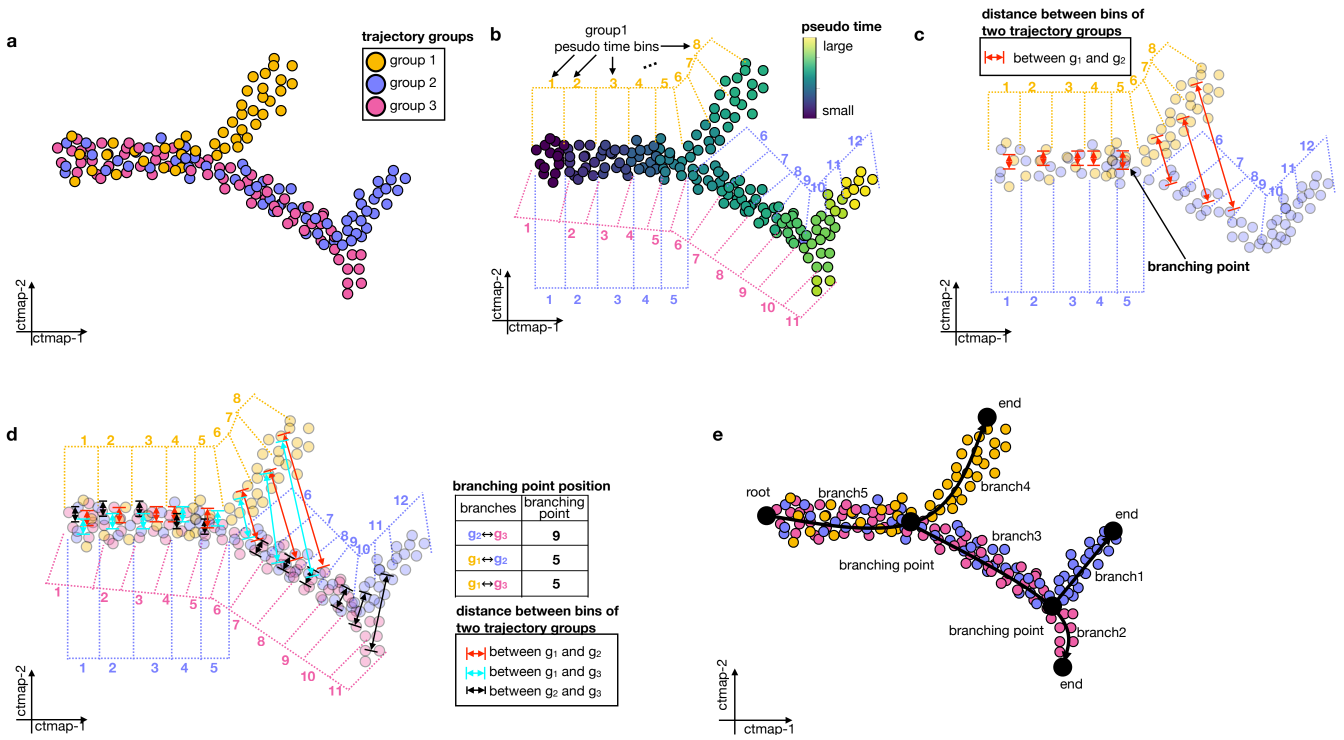

Figure S10. Schematic showing how to create a tree using edges lay in cumulative trajectory space. a, Example of three trajectory groups g_1, g_2, g_3 each with a distinct color in the cumulative trajectory space. Every dot in this embedding represents an edge in the graph (or a cell differentiation event). **b,** PHLOWER takes the mean on the pseudo-time of the vertices (cells) connected by an edge and uses this as the edge pseudo-time estimate. PHLOWER next bins the edges by considering their pseudo-times. **c,** It then estimates average edge locations for every bin, which serves as a backbone for the trajectories. It also estimates the distance between edges in two groups but with the same bin index. **d,** Distance between bins are estimated for all groups and branchings points are detected whenever the distance between edges in two groups surpasses the distances of edges within a group for a given bin In the example, bin 9 is a branching point between groups 2 and 3 and bin 5 is a branching point between groups 1 and 2 and 1 and 3. **e,** To build the final tree, PHLOWER considers the branching points with the highest values towards the lowest values to build trees in a bottom-up approach, i.e. it merges trajectory 2 and 3 at bin 9. The next branching point is 5, which connects trajectory 1 with the tree formed by 2 and 3.

name	structure type	nbranch	ncells	standard	nb/branch	criterion
**planaria-full_plass**	tree	26	18837	silver	724.5	✓
**planaria-pair-13_plass**	tree	16	13265	silver	829.1	✓
**planaria-pair-9_plass**	tree	13	11319	silver	870.7	✓
**planaria-pair-2_plass**	tree	12	10653	silver	887.8	✓
**planaria-pair-12_plass**	tree	11	11685	silver	1062.3	✓
**planaria-pair-7_plass**	tree	10	11009	silver	1100.9	✓
**ICM-monkey_nakamura**	tree	9	272	silver	30.2	✓
**planaria-pair-10_plass**	tree	9	14636	silver	1626.2	✓
**planaria-pair-14_plass**	tree	8	13281	silver	1660.1	✓
**planaria-pair-4_plass**	tree	8	8960	silver	1120.0	✓
**fetal-liver-fetal-hematopoiesis_mca**	multifurcation	7	2559	silver	365.6	✓
**oligodendrocyte-differentiation-subclusters_marques**	multifurcation	7	4930	silver	704.3	✓
**planaria-pair-11_plass**	tree	7	12978	silver	1854.0	✓
**epiblast-monkey_nakamura**	tree	6	182	silver	30.3	✓
**hematopoiesis-clusters_olsson**	tree	6	376	silver	62.7	✓
**planaria-neuron-differentiation_plass**	multifurcation	6	2349	silver	391.5	✓
**planaria-pair-3_plass**	tree	6	8589	silver	1431.5	✓
**planaria-pair-6_plass**	tree	6	8983	silver	1497.2	✓
**embronic-mesenchyme-neuron-differentiation_mca**	tree	5	481	silver	96.2	✓
**mesoderm-development_loh**	tree	4	504	gold	126.0	✓
**planaria-pair-5_plass**	tree	4	12660	silver	3165.0	✓
**planaria-pair-8_plass**	tree	4	9354	silver	2338.5	✓
**planaria-parenchyme-differentiation_plass**	multifurcation	4	1986	silver	496.5	✓
**germline-human-both_guo**	bifurcation	3	272	gold	90.7	✓
**macrophage-salmonella_saliba**	multifurcation	3	60	gold	20.0	✓
**NKT-differentiation_engel**	multifurcation	3	197	gold	65.7	✓
**cortical-interneuron-differentiation_frazer**	multifurcation	3	213	silver	71.0	✓
**fibroblast-reprogramming_treutlein**	bifurcation	3	355	silver	118.3	✓
**hepatoblast-differentiation_yang**	bifurcation	3	504	silver	168.0	✓
**kidney-collecting-duct-subclusters_park**	bifurcation	3	1533	silver	511.0	✓
**neonatal-rib-cartilage_mca**	bifurcation	3	2221	silver	740.3	✓
**placenta-trophoblast-differentiation_mca**	multifurcation	3	1001	silver	333.7	✓
**trophoblast-stem-cell-trophoblast-differentiation_mca**	multifurcation	3	19467	silver	6489.0	✓
distal-lung-epithelium_treutlein	bifurcation	2	59	silver	29.5	✗ simple
planaria-muscle-differentiation_plass	bifurcation	2	2338	silver	1169.0	✗ simple
planaria-pair-1_plass	bifurcation	2	10578	silver	5289.0	✗ simple
thymus-t-cell-differentiation_mca	bifurcation	2	1607	silver	803.5	✗ simple
aging-hsc-old_kowalczyk	linear	1	873	gold	873.0	✗ simple
aging-hsc-young_kowalczyk	linear	1	493	gold	493.0	✗ simple
developing-dendritic-cells_schlitzer	linear	1	238	gold	238.0	✗ simple
germline-human-female_guo	linear	1	100	gold	100.0	✗ simple
germline-human-female-weeks_li	linear	1	666	gold	666.0	✗ simple
germline-human-male_guo	linear	1	166	gold	166.0	✗ simple
germline-human-male-weeks_li	linear	1	649	gold	649.0	✗ simple
hematopoiesis-gates_olsson	linear	1	316	gold	316.0	✗ simple
human-embryos_petropoulos	linear	1	1289	gold	1289.0	✗ simple
mESC-differentiation_hayashi	linear	1	414	gold	414.0	✗ simple
myoblast-differentiation_trapnell	linear	1	290	gold	290.0	✗ simple
pancreatic-alpha-cell-maturation_zhang	linear	1	322	gold	322.0	✗ simple
pancreatic-beta-cell-maturation_zhang	linear	1	562	gold	562.0	✗ simple
psc-astrocyte-maturation-glia_sloan	linear	1	455	gold	455.0	✗ simple
psc-astrocyte-maturation-neuron_sloan	linear	1	192	gold	192.0	✗ simple
stimulated-dendritic-cells-LPS_shalek	linear	1	540	gold	540.0	✗ simple
stimulated-dendritic-cells-PAM_shalek	linear	1	407	gold	407.0	✗ simple
stimulated-dendritic-cells-PIC_shalek	linear	1	433	gold	433.0	✗ simple

Table S1. List of all Dynverse data sets with a tree structure sorted by the number of branches. We only considered data sets (in bold) with at least 3 branches.

names	celltype	Source
ESRP1	Tubular	Computational Gene Markers
BLNK	Tubular	Computational Gene Markers
DCDC2	Tubular	Computational Gene Markers
MACC1	Tubular	Computational Gene Markers
PAX2	Tubular	Computational Gene Markers
LHX1	Tubular	Computational Gene Markers
RASSF6	Tubular	Computational Gene Markers
EMX2	Tubular	Computational Gene Markers
CACNA2D3	Tubular	Computational Gene Markers
GRHL2	Tubular	Computational Gene Markers
ERBB4	Tubular	Computational Gene Markers
POU3F3	Tubular	Computational Gene Markers
NPHS2	Podocytes	Computational Gene Markers
CLIC5	Podocytes	Computational Gene Markers
ZBTB7C	Podocytes	Computational Gene Markers
WT1	Podocytes	Computational Gene Markers
NPHS1	Podocytes	Computational Gene Markers
PODXL	Podocytes	Computational Gene Markers
NRG3	Podocytes	Computational Gene Markers
PTPRO	Podocytes	Computational Gene Markers
MAFB	Podocytes	Computational Gene Markers
TESC	Podocytes	Computational Gene Markers
CPEB1	Podocytes	Computational Gene Markers
FRY	Podocytes	Computational Gene Markers
GPC5	Stromal1	Computational Gene Markers
L1TD1	Stromal1	Computational Gene Markers
TWIST1	Stromal1	Computational Gene Markers
SH2D3C	Stromal1	Computational Gene Markers
EDNRA	Stromal1	Computational Gene Markers
LAMA4	Stromal1	Computational Gene Markers
WNT5B	Stromal1	Computational Gene Markers
SHOX2	Stromal234	Computational Gene Markers
RSPO2	Stromal234	Computational Gene Markers
BEND4	Stromal234	Computational Gene Markers
MKX	Stromal234	Computational Gene Markers
LHX9	Stromal234	Computational Gene Markers
PTGFR	Stromal234	Computational Gene Markers
DLX2	Stromal234	Computational Gene Markers
GABRR1	Stromal234	Computational Gene Markers
FGF14	Stromal234	Computational Gene Markers
CDH12	Stromal234	Computational Gene Markers
CHRM3	Stromal234	Computational Gene Markers
FRMPD4	Stromal234	Computational Gene Markers
LMX1A	Neuron12	Computational Gene Markers
WSCD2	Neuron12	Computational Gene Markers
RFX4	Neuron12	Computational Gene Markers
WNT10B	Neuron12	Computational Gene Markers
RAB3C	Neuron12	Computational Gene Markers
FYB2	Neuron12	Computational Gene Markers
TMEM255A	Neuron12	Computational Gene Markers

names	celltype	Source
CR1L	Neuron12	Computational Gene Markers
EDAR	Neuron12	Computational Gene Markers
DCC	Neuron12	Computational Gene Markers
WNT1	Neuron12	Computational Gene Markers
CDH6	Neuron12	Computational Gene Markers
SMPX	Muscle	Computational Gene Markers
IGSF21	Muscle	Computational Gene Markers
HTR2C	Muscle	Computational Gene Markers
PAX7	Muscle	Computational Gene Markers
MYO18B	Muscle	Computational Gene Markers
FRMPD1	Muscle	Computational Gene Markers
MYF6	Muscle	Computational Gene Markers
MYL1	Muscle	Computational Gene Markers
CELF2	Muscle	Computational Gene Markers
MRLN	Muscle	Computational Gene Markers
POU4F1	Muscle	Computational Gene Markers
RGS7	Muscle	Computational Gene Markers
FOXC2	Tubular	Transcription factors
FOXP1	Tubular	Transcription factors
FOXO3	Tubular	Transcription factors
MAF	Tubular	Transcription factors
HNF1B	Tubular	Transcription factors
MAFG	Podocytes	Transcription factors
ERF	Stromal1	Transcription factors
CLOCK	Stromal1	Transcription factors
ETV5	Stromal1	Transcription factors
MAX	Stromal1	Transcription factors
FLI1	Stromal1	Transcription factors
NFIA	Stromal234	Transcription factors
NFAT5	Stromal234	Transcription factors
RUNX1	Stromal234	Transcription factors
NFATC4	Stromal234	Transcription factors
NFIB	Stromal234	Transcription factors
RUNX2	Stromal234	Transcription factors
PAX3	Neuron12	Transcription factors
HOXB8	Neuron12	Transcription factors
LEF1	Neuron12	Transcription factors
ZIC2	Neuron12	Transcription factors
HOXC10	Neuron12	Transcription factors
MYOG	Muscle	Transcription factors
MSC	Muscle	Transcription factors
MYF5	Muscle	Transcription factors
TBXT	Mesorderm	Literatuer Cell Markers
KDR	Mesorderm	Literatuer Cell Markers
PAPPA2	Tubular	Literatuer Cell Markers
SLC12A1	Tubular	Literatuer Cell Markers
MSX1	Neuron	Literatuer Cell Markers
MAP2	Neuron	Literatuer Cell Markers
PDGFRB	Stromal	Literatuer Cell Markers
COL1A2	Stromal	Literatuer Cell Markers

Table S2. List of genes used in the Xenium spatial profiling panel. Source indicates if marker were based on computational markers from the mulitome data; literature markers or TFs predicted by PHLOWER and scMEGA.

References

1. Bergen, V., Lange, M., Peidli, S., Wolf, F. A. & Theis, F. J. Generalizing rna velocity to transient cell states through dynamical modeling. *Nat. Biotechnol.* **38**, 1408–1414, DOI: 10.1038/s41587-020-0591-3 (2020).

2. La Manno, G. *et al.* RNA velocity of single cells. *Nature* **560**, 494–498, DOI: 10.1038/s41586-018-0414-6 (2018).

3. Frantzen, F., Seby, J.-B. & Schaub, M. T. Outlier detection for trajectories via flow-embeddings. In *2021 55th Asilomar Conference on Signals, Systems, and Computers*, 1568–1572, DOI: 10.1109/ieeeconf53345.2021.9723128 (IEEE, 2021).
