# Supplementary material for: PHLOWER leverages single-cell multimodal data to infer complex, multi-branching cell differentiation trajectories
Source: Nat Methods. 2025 Oct 23;22(11):2328–36. doi: 10.1038/s41592-025-02870-5 (PMC12615267; doi:10.1038/s41592-025-02870-5)
Supplement: Supplementary file 2 — Reporting Summary [file 41592_2025_2870_MOESM2_ESM.pdf]

nature portfolio

Corresponding author(s): Ivan G. Costa

Last updated by author(s): Jun 3, 2025

Reporting Summary

Nature Portfolio wishes to improve the reproducibility of the work that we publish. This form provides structure for consistency and transparency in reporting. For further information on Nature Portfolio policies, see our Editorial Policies and the Editorial Policy Checklist.

Statistics

For all statistical analyses, confirm that the following items are present in the figure legend, table legend, main text, or Methods section.

n/a	Confirmed	
☐	☒	The exact sample size (*n*) for each experimental group/condition, given as a discrete number and unit of measurement
☐	☒	A statement on whether measurements were taken from distinct samples or whether the same sample was measured repeatedly
☐	☒	The statistical test(s) used AND whether they are one- or two-sided<br>*Only common tests should be described solely by name; describe more complex techniques in the Methods section.*
☐	☒	A description of all covariates tested
☐	☒	A description of any assumptions or corrections, such as tests of normality and adjustment for multiple comparisons
☐	☒	A full description of the statistical parameters including central tendency (e.g. means) or other basic estimates (e.g. regression coefficient) AND variation (e.g. standard deviation) or associated estimates of uncertainty (e.g. confidence intervals)
☐	☒	For null hypothesis testing, the test statistic (e.g. *F*, *t*, *r*) with confidence intervals, effect sizes, degrees of freedom and *P* value noted<br>*Give P values as exact values whenever suitable.*
☒	☐	For Bayesian analysis, information on the choice of priors and Markov chain Monte Carlo settings
☒	☐	For hierarchical and complex designs, identification of the appropriate level for tests and full reporting of outcomes
☒	☐	Estimates of effect sizes (e.g. Cohen's *d*, Pearson's *r*), indicating how they were calculated

Our web collection on statistics for biologists contains articles on many of the points above.

Software and code

Policy information about availability of computer code

Data collection

no software was used for data collection.

Data analysis

We provide a Python package `PHLOWER`available at https://github.com/CostaLab/phlower/. All the trajectory inference and regulator discovery functions are implemented in `PHLOWER` package which can be installed by `git clone https://github.com/CostaLab/phlower/; cd phlower; pip install .`. We also built a website (https://phlower.readthedocs.io) where we provide the installation guide as well as tutorial of dataset `fib2neuron` and `kidney` we generated and presented in this manuscript. We also provide the benchmarking data as well as the multiome kidney organoid and xenium kidney organoid data presented in the manscript in zenodo:https://doi.org/10.5281/zenodo.13860460

Tools used in the preprocessing in the manuscript for multiome analysis:

R:
R v4.1.3
Seurat v3.2.3
ArchR v1.0.2
MOJITOO v1.0.0
Signac v1.9.0
chromVAR v1.20.0
DropletUtils v1.18.1
dplyr v1.1.2
ggplot2 v3.4.2

SingleCellExperiment 1.20.0

Tools used in the preprocessing in the manuscript for xenium analysis:
Seurat v5.0.2
ggplot2 v3.5.1
dplyr v1.1.4

Tools used in the data analysis in the manuscript for phlower:
Python:
Python v3.10.8
anndata v0.9.2
colorcet v3.0.1
matplotlib v3.9.1
networkx v2.8.8
numpy v1.23.5
pandas v2.2.3
pydot v1.4.2
scanpy v1.9.3
scipy v1.14.0
seaborn v0.13.2
sklearn v1.5.1

For manuscripts utilizing custom algorithms or software that are central to the research but not yet described in published literature, software must be made available to editors and reviewers. We strongly encourage code deposition in a community repository (e.g. GitHub). See the Nature Portfolio guidelines for submitting code & software for further information.

Data

Policy information about availability of data

All manuscripts must include a data availability statement. This statement should provide the following information, where applicable:

- Accession codes, unique identifiers, or web links for publicly available datasets
- A description of any restrictions on data availability
- For clinical datasets or third party data, please ensure that the statement adheres to our policy

All pre-processed single cell and spatial data sets were deposited in zenodo (https://doi.org/10.5281/zenodo.13860460). Raw sequencing files have been deposited in GEO (GSE302266, GSE302264).

Human research participants

Policy information about studies involving human research participants and Sex and Gender in Research.

Reporting on sex and gender	N.A.
Population characteristics	N.A.
Recruitment	N.A.
Ethics oversight	N.A.

Note that full information on the approval of the study protocol must also be provided in the manuscript.

Field-specific reporting

Please select the one below that is the best fit for your research. If you are not sure, read the appropriate sections before making your selection.

☒ Life sciences ☐ Behavioural & social sciences ☐ Ecological, evolutionary & environmental sciences

For a reference copy of the document with all sections, see nature.com/documents/nr-reporting-summary-flat.pdf

Life sciences study design

All studies must disclose on these points even when the disclosure is negative.

Sample size	At least n=3 were used per organoid. No data point was excluded.
Data exclusions	N.A.

Replication	N.A.
Randomization	N.A.
Blinding	No blinding.

Reporting for specific materials, systems and methods

We require information from authors about some types of materials, experimental systems and methods used in many studies. Here, indicate whether each material, system or method listed is relevant to your study. If you are not sure if a list item applies to your research, read the appropriate section before selecting a response.

Materials & experimental systems

n/a	Involved in the study	
☐	☒	Antibodies
☐	☒	Eukaryotic cell lines
☒	☐	Palaeontology and archaeology
☒	☐	Animals and other organisms
☒	☐	Clinical data
☒	☐	Dual use research of concern

Methods

n/a	Involved in the study	
☒	☐	ChIP-seq
☒	☐	Flow cytometry
☒	☐	MRI-based neuroimaging

Antibodies

Antibodies used	Primary antibodies: (NPHS1, catnr AF 4269-SP, RD Systems. Vimentin, catnr ab92547, Abcam. E-cadherin, catnr 610405, BD Biosciences. Secondary antibodies: donkey anti-sheep IgG (H+L) alexa fluor 647 (ThermoFisher), donkey anti-rabbit IgG (H+L) alexa fluor 488 (ThermoFisher), donkey anti-mouse IgG (H+L) alexa fluor 488 (ThermoFisher), DAPI (Sigma Aldrich, D9542) Merck).
Validation	The antibodies were validated in a previous study using human kidney tissue. Please refer to Jansen et al Cell Stem Cell 2022 doi: 10.1016/j.stem.2021.12.010, Jansen et al Develoment 2022 doi: 10.1242/dev.200198 and Takasato et al Nature 2015 doi.org/10.1038/nature15695.

Eukaryotic cell lines

Policy information about cell lines and Sex and Gender in Research

Cell line source(s)	The iPS cells were reprogrammed from human somatic cells obtained from a healthy volunteer after given informed consent.
Authentication	no authentication was performed.
Mycoplasma contamination	The cells were tested negative for mycoplasma.
Commonly misidentified lines (See ICLAC register)	n.a.
